# Estimation of carcass weight of Hanwoo (Korean native cattle) as a function of body measurements using statistical models and a neural network

**DOI:** 10.5713/ajas.19.0748

**Published:** 2019-12-24

**Authors:** Dae-Hyun Lee, Seung-Hyun Lee, Byoung-Kwan Cho, Collins Wakholi, Young-Wook Seo, Soo-Hyun Cho, Tae-Hwan Kang, Wang-Hee Lee

**Affiliations:** 1 Department of Biosystems Machinery Engineering, Collage of Agricultural and Life Science, Chungnam National University, Daejeon 34134, Korea; 2 National Institute of Agricultural Sciences, Rural Development Administration, Jeonju 54875, Korea; 3 Animal Products Utilization Division, National Institute of Animal Science, Rural Development Administration, Wanju 55365, Korea; 4 Major in Bio-Industry Mechanical Engineering, Kongju National University, Yesan 32439, Korea

**Keywords:** Body Measurement, Carcass Weight, Hanwoo, Multiple Regression, Partial Least Square Regression, Neural Network

## Abstract

**Objective:**

The objective of this study was to develop a model for estimating the carcass weight of Hanwoo cattle as a function of body measurements using three different modeling approaches: i) multiple regression analysis, ii) partial least square regression analysis, and iii) a neural network.

**Methods:**

Data from a total of 134 Hanwoo cattle were obtained from the National Institute of Animal Science in South Korea. Among the 372 variables in the raw data, 20 variables related to carcass weight and body measurements were extracted to use in multiple regression, partial least square regression, and an artificial neural network to estimate the cold carcass weight of Hanwoo cattle by any of seven body measurements significantly related to carcass weight or by all 19 body measurement variables. For developing and training the model, 100 data points were used, whereas the 34 remaining data points were used to test the model estimation.

**Results:**

The R^2^ values from testing the developed models by multiple regression, partial least square regression, and an artificial neural network with seven significant variables were 0.91, 0.91, and 0.92, respectively, whereas all the methods exhibited similar R^2^ values of approximately 0.93 with all 19 body measurement variables. In addition, relative errors were within 4%, suggesting that the developed model was reliable in estimating Hanwoo cattle carcass weight. The neural network exhibited the highest accuracy.

**Conclusion:**

The developed model was applicable for estimating Hanwoo cattle carcass weight using body measurements. Because the procedure and required variables could differ according to the type of model, it was necessary to select the best model suitable for the system with which to calculate the model.

## INTRODUCTION

Body weight of beef cattle is one of the most important traits affecting price [1] and animal condition [2,3]. For this reason, accurate estimation of body weight is emphasized to establish adequate management and nutritional approaches for improving conditions for raising beef cattle and maximizing profits [4]. Because body weight is related to the body size of beef cattle, body size measurement is considered the main physical estimator of body weight [5,6]. Unlike internal traits, such as body composition and genetic characteristics [7], body size is easy to measure; thus, it has been used to evaluate body weight [8–10].

Body weight of beef cattle has been estimated as a function of body measurements according to cattle species, age, and gender. In particular, as image analysis in automated carcass weight measurement has been demanded by the livestock industry, a simple model for predicting body weight has been coded as an algorithm [10]. Heinrichs et al [8] predicted the body weight of Holstein heifers through body measurements based on a large number of observations and found a greater than 95% R^2^-value. Ozkaya and Bozkurt [5] applied regression analysis to predict body weight from body measurements in Holstein, Brown Swiss, and crossbred cattle with R^2^ values of 92%, 95%, and 68%, respectively. For Holstein–Friesian lactating dairy cows, body size measurements were used to estimate live weight (78%), empty body weight (75%), and carcass weight (75%) but model accuracy was relatively low [4]. Haryoko and Suparman [11] used multiple regression to assess carcass weight according to rear girth, body condition, and slaughter weight. Tasdemir et al [10] developed a regression model to estimate the body weight of Holstein cows by determining body measurements using digital image analysis (maximum of 98% accuracy).

Preference for Hanwoo, Korean native cattle breed, has increased in South Korea because of food safety concerns and its unique taste [12]. Carcass yield is the factor affecting profit in the livestock industry and distributional policy in the government, along with meat quality [13,14]. Thus, carcass weight, which is a direct outcome from body weight and size of the carcass are the main variables measured. However, grading and measurements of Hanwoo cattle are still dependent on human judgment, necessitating a digital tool for assisting in the judgment. For this, it is critical to develop a predictive model that evaluates carcass weight by body measurements as a basic algorithm implemented in a system. There have been a few studies regarding Hanwoo cattle using body size traits to estimate beef cut yield [15,16], estimate carcass yield [17], and classify body type [18,19]. However, for Hanwoo cattle, prediction of carcass weight by body measurements has not been studied, although a predictive model must differ by cattle species.

Recent applications of statistics-based machine learning and deep learning on agricultural data has allowed the development of predictive models with high accuracy compared to traditional approaches. This suggests that this state-of-the-art technology may be applicable to the development of a model for estimating body or carcass weight using variables that could be easily measured either by humans or a digital machine. This study, therefore, estimated carcass weight of Hanwoo cattle as a function of body measurements using 3 different approaches, multiple regression, partial least square (PLS) regression, and a neural network, and compared their abilities to identify the optimal methodology to estimate carcass weight. Because a simple way to estimate carcass weight has been emphasized for establishing a national demand/supply policy and for embedding an algorithm into digital imaging systems, we expect this study will provide the best predictive model for Hanwoo cattle, regarding of the aforementioned necessities.

## MATERIALS AND METHODS

### Animal data

The raw data were obtained from the National Institute of Animal Science (NIAS) in South Korea, which contained pre-deboning and post-deboning variables for Hanwoo cattle, such as carcass weight, weights for primal cuts, and quality indices. The age of Hanwoo cattle ranged from 17 to 120 months (mean of 41±18.6 months), and they were slaughtered from 2016 to 2018 in various sites in South Korea. The total number of Hanwoo cattle in the data was 134, consisting of 24 bulls, 49 cows, and 61 steers.

### Body measurement data

From among a total of 372 variables in the raw data, we extracted 20 variables related to body size measurement, because this study focused on the prediction of carcass weight according to this type of data. These included cold carcass weight (CWT, kg), backfat thickness (FT, mm), eye muscle area (EMA, cm^2^), side length (L, cm), forequarter length (LF, cm), hindquarter length (LB, cm), cervical vertebrae length (L1, cm), thoracic vertebrae length (L2, cm), lumbar vertebrae length (L3, cm), sacral vertebrae length (L4, cm), 6th lumbar vertebrae-heel length (L5, cm), 7th cervical vertebrae carcass breadth (L6, cm), 5–6th thoracic vertebrae breadth (L7, cm), 4–5th lumbar vertebrae breadth (L8, cm), 5th sacral vertebrae breadth (L9, cm), 7–8th thoracic vertebrae girth (L10, cm), coxae girth (chest girth, L11, cm), 4–5th lumbar vertebrae thick (L12, cm), coxae thick (L13, cm), and 7–8th thoracic vertebrae thick (L14, cm). The definitions of body size measurement were taken from Kim et al [20]. All 20 variables are summarized in Table 1.

### Multiple linear regression analysis

Multiple linear regression analysis was used to develop a correlative model that contained more than one explanatory variable, with the general formula in equation 1 [21]:

(Equation 1) $$Y=\beta_{0}+\beta_{1}x_{1}+\beta_{2}x_{2}+\ldots \beta_{\text{n}}x_{\text{n}}+\in ,$$

where *Y* is the dependent variable to be estimated, *x*_i_ represents an independent (or explanatory) variable, and *∈* is a random error term. *β*_0_ represents the intercept, whereas *β*_i_ is the regression coefficient.

Because of its simplicity in model development, it has been applied to estimate body weight of beef cattle as a function of body size for other cattle breeds [9,10]. In this study, CWT was the dependent variable, while the other 19 variables related to body measurement were considered potential explanatory variables. In the modeling, we firstly used all 19 variables and then selected the significant variables based on estimated p-values that were <0.05. Then, the significant variables were used to develop a model with 100 data points, whereas the remaining 34 data points were used to validate the model by calculating relative error (Equation 2). Model accuracy was judged based on the R^2^ value of the developed model and the average relative error from model validation.

(Equation 2) $$Relative\;error=\left|\frac{measured\;weight-estimated\;weight}{measured\;weight}\right|$$

### Multivariate analysis: partial least square regression

The PLS regression is a generalized multiple linear regression model for handling multicollinearity among variables [22]. This method develops a new set of uncorrelated variables by linearly combining the original variables and reduces dimensionality to maximize the explained variances of the independent variables without considering the correlations among independent variables and responses [23]. Because of this ability, PLS regression is widely used as a basic tool in chemometrics to analyze spectral and imaging data from agricultural and livestock products [24]. Because PLS regression is a statistics-based machine-learning approach, we separated the entire dataset into training (100 data points) and test datasets (34 data points) for developing and testing the model, respectively. Model accuracy was determined based on the percent of explained variance in the dependent variable, i.e., CWT, and relative error calculated by Equation 2.

### Neural network

An artificial neural network (ANN) can enhance the accuracy in regression and classification tasks by learning data and mathematically mimicking the human brain structure of interconnected neurons [25,26]. The general layout of an ANN has an input layer, a hidden layer, and an output layer, which are functionally connected to each other. Regarding the regression analysis, the neural network is trained through supervised learning methods that update the weights between nodes (neurons) with labeled data consisting of input variables to desired output value pairs. The error between the predicted output from the ANN and the desired output, i.e., the cost, is used to train the model, and the weight of each layer is updated by the gradient descent update rule with a back-propagation algorithm to minimize the error. In general, larger numbers of hidden layers generally provide higher training accuracy, but it is also at risk of over-fitting [27]. In other words, more hidden layers can train the ANN more appropriate to the training data while it decreases prediction accuracy for the test data, losing its generalization ability [26]. In this study, we firstly determined the number of hidden layers and the number of neurons in each hidden layer to construct the ANN for estimation of carcass weight. The hidden layer has two stages, and each stage has 15 and 6 neurons, respectively. The ANN model was repeatedly trained and validated at every training iteration. The training was stopped earlier than the minimum validation cost to avoid over-fitting caused by too many trainings. The selected significant variables and the CWT were used as a pair of training data, and 100 and 34 data points were used for network training and validation, respectively. The mean squared error was used as a cost function and weights were updated using a stochastic gradient descent optimizer with 0.9 momenta. The learning rate was set at 0.01, reducing the rate to 10% per 100 iterations.

### Software

All statistical analyses were performed using the SAS software package (ver. 9.4, SAS Institute Inc., Cary, NC, USA). Statistical significance was assumed when the p-value was less than 0.05. For the ANN, Python programming language (ver. 3.6, Python Software Foundation, Beaverton, OR, USA) was used to implement the environment of machine learning and the TensorFlow python library (ver. 1.13.1, Google, CA, USA) was used to build the neural network and train it.

## RESULTS AND DISCUSSION

### Variable selection

To select variables for explaining the carcass weight in the model, we firstly investigated the correlation of CWT with size measurements using the Pearson correlation coefficient (Table 2). As expected, high correlations between variables were observed because weight and size are known to be related [17]. In particular, all the size measurement variables were significantly correlated with carcass weight, indicating they could be candidates for explanatory variables in the predictive model. In detail, L was correlated with all other variables, including CWT except FT (r = −0.081, p = 0.357), which was consistent with the results of a study on body length, i.e., L in our data, as an indicator of body weight in beef cattle [5,10]. Our analysis also showed that coxae girth (chest girth, L11) was correlated with all other variables, consistent with the results of a study wherein it was used as the main determinant in estimating the body weight of beef cattle [9,15]. In addition, carcass weight was shown to have significant correlations with FT, EMA, and L11 in Hanwoo cattle [17], suggesting that these variables could be potential explanatory variables. In our analysis, EMA exhibited a significant correlation with CWT, but it was not correlated with FT (r = −0.031, p = 0.728). Because of multicollinearity, the explanatory variables in the model should be independent [22], whereas most of variables in this study were correlated with each other. In this point of view, it was preferable to include EMA in the model as an explanatory variable.

### Estimation of carcass weight using the multiple regression model

**Figure 1:**
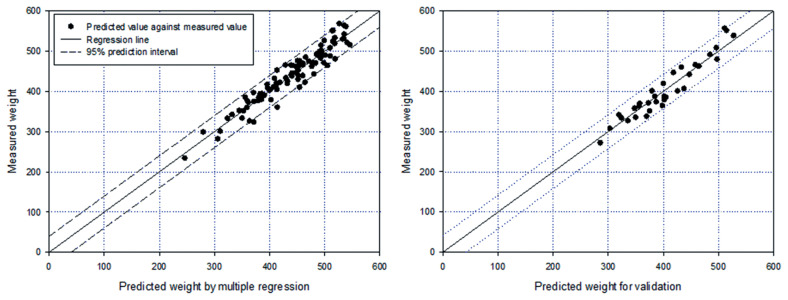
Results of multiple linear regression by comparing predicted values against measured weights. (A) Model development result (R^2^ = 0.91) and (B) model validation result (R^2^ = 0.91).

As an initial attempt, we employed a multiple regression model without considering multicollinearity. When including all the variables, the R^2^ of the model was 0.93, but 11 variables (L1–L5, L7–L9, and L12–L14) were not significant, suggesting that we needed to remove them from the model. To develop a model with significant variables (L, FT, EMA, L6, L11, and L12) we used 100 data point, while the 34 remaining were used to validate the model predictions. As a result, the developed model exhibited an R^2^ of 0.91 and residuals were randomly distributed (p-value = 0.115 in the constant variance test), indicating the model prediction was reliable and linear structure of the model was adequate (Figure 1 and Equation 3). In addition, the significant variables in the model were similar to that of a previous analysis of Hanwoo cattle [17,18], and chest girth was the common factor in predicting body weight by body measurements in other species [9].

(Equation 3) CWT=-805.6+1.16FT+1.16EMA+1.36L+3.32L6+1.53L10+1.79L11

With the 34 data points that were not used for model development, the average relative error was calculated using equation 2. It was approximately 4% with minimum and maximum errors of 4% and 10%, respectively. The largest error was observed in a bull having the least weight among bulls and had relatively less FT but a higher coxae girth compared to cows, which had a similar carcass weight. Practically, body size variables are independently measured by judgement, suggesting the explanatory variables used in the model are practically independent. Thus, the model is applicable for estimating carcass weight of Hanwoo cattle. However, from a statistical point of view, this model did not consider the problem caused by multicollinearity because the significant explanatory variables used in the model were correlated with each other. In addition, none of the previous models considered this issue. Consequently, it is necessary to use multivariate analysis to produce a new set of variables, which were not correlated and compare models with and without considering multicollinearity.

### Estimation of carcass weight using partial least square regression

**Figure 2:**
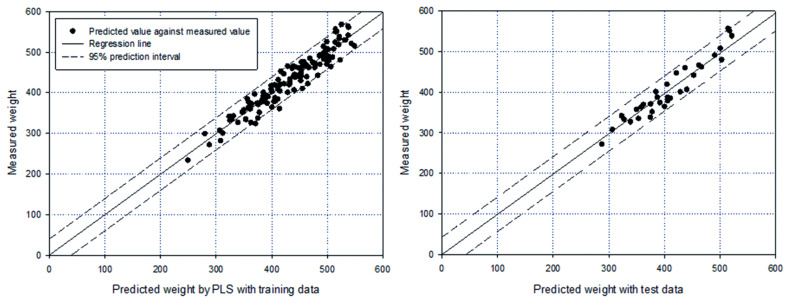
Results of partial least square modeling by comparing predicted values against measured weights with (A) training data (R^2^ = 0.92) and (B) test data (R^2^ = 0.91).

As mentioned above, multicollinearity may cause inaccurate estimation of regression coefficients in the model because of the interdependency between explanatory variables. In our correlation analysis, it was shown that all the measurements, except FT, were correlated with each other, suggesting the necessity of considering multicollinearity. For this reason, we used PLS regression to avoid the multicollinearity problem [22,23]. As the first step, a principal component analysis (PCA) was used to identify the significant explanatory variables. The results showed that the first 4 principal components were able to explain approximately 65% of the variance in all explanatory variables and eigenvalues were greater than 1, suggesting these components were critical for explaining the variance [28]. In the first 4 principal components, FT, L, L2, L3, L4, L6, L7, L10, L11, L12, and L13 exhibited greater values for eigenvectors; thus, they could be the main components in the PLS model. With comprehensive consideration of the results of the PCA and multiple regression analysis, we selected FT, EMA, L, L6, L10, and L11, which were the same variables in the previous regression model, for use in the PLS regression. In the PLS regression, we used 100 and 34 data points for training and test datasets, respectively. As a result, the developed model was able to explain 91% of the variation in CWT, which was similar to the model accuracy of the regression model (Figure 2). The result also was not significantly lower than the R^2^ with all 19 variables (R^2^ = 0.92). The intercept and coefficients showed very similar values compared to those in the multiple regression model, suggesting the applicability of both models to estimate carcass weight (Equation 4).

(Equation 4) CWT=-808.9+1.03FT+1.18EMA+1.26L+3.06L6+1.79L10+1.85L11

With the test dataset, the relative error was approximately 4%, which was the same as that of the multiple regression model, and the minimum and maximum errors were 1% and 11%, respectively. The largest error was shown in the same individual, which had the largest error in the multiple regression model.

### Estimation of carcass weight using a neural network

**Figure 3:**
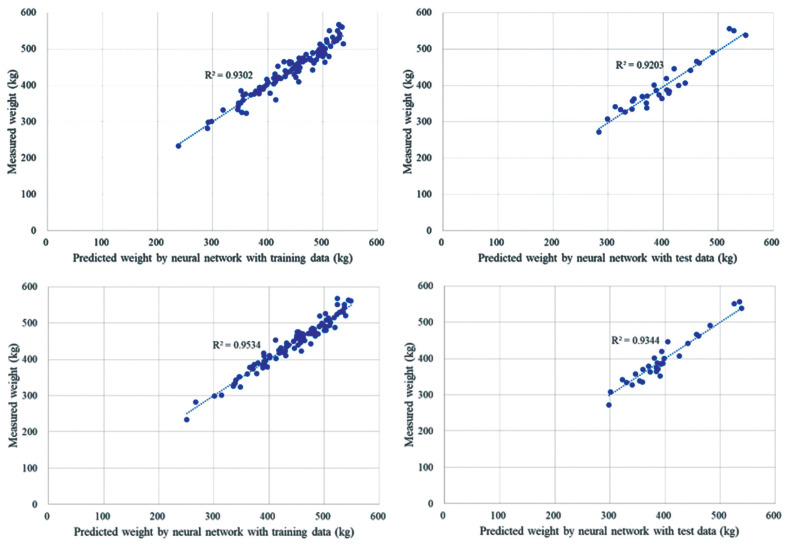
Result of artificial neural network by comparing predicted value against measured weight by using (A) 6 variables and (B) all 19 variables with (left) with training data, and (right) with test data.

During the performance of deep learning by the constructed ANN to estimate CWT, two different groups of variables were used; six significant variables (L, FT, EMA, L6, L11, and L12) and all 19 variables of body measurements. The validation cost was evaluated for every training iteration and the weights with the lowest validation cost were used to predict CWT. When the ANN was trained with the 6 significant variables (L, FT, EMA, L6, L11, and L12), it stopped at 1,192 iterations and exhibited the lowest cost. The prediction showed an R^2^ of 0.92, which was slightly higher than that of the multiple regression and PLS models and the relative error was approximately 4% (Figure 3A). With 19 variables, the predicted CWT was highly correlated with the CWT measured with the training data (R^2^ = 0.95), and the model predicted the CWT with an R^2^ of 0.93 using the test data with a mean relative error of 4% (Figure 3B). Compared to the statistics-based models, R^2^ was higher by 1% but the relative error was not improved. Furthermore, we added age and gender to the 19 variables of body measurements and performed the ANN with a total of 21 variables. When training the networks without considering validation cost, the ANN could improve the R^2^ up to 0.98 for the training data. However, the R^2^ for the test data decreased to 0.85 because of over-fitting. Considering the validation cost, it had the lowest value at 1,334 iterations, and the R^2^ value was 0.94 for both the training and test datasets. Therefore, the ANN model with 21 variables showed the best performance in the estimation of CWT compared to the other models developed in this study. This suggested that the addition of the 2 variables, i.e., gender and age, could enhance the correlation between input and output variables in the ANN-based approach. Despite the high correlation shown above, the performance of the ANN modeling was dependent on initial weights of the network. The ANN could easily be saturated by local minima or overfitting when the initial weights were generated with excessive cost or bias in training data. In this study, the ANN models were developed using a total of 134 data points, which small compared to previous ANN-based approaches. To train the ANN to have a higher generalization ability without overfitting, it is effective to increase the number of training data and optimize the network architecture.

### Discussion regarding the selection of the optimal model

The developed model is a useful algorithm for an automated machine system which rapidly measures only a few carcass size variables in the image [10]. Among the developed models, multiple regression analysis was the simplest, and it effectively represented the power of a correlative model with only significant variables. By reducing the number of variables to be practically measured (only 6 variables), the multiple regression model was a simple way to estimate CWT. However, as previously mentioned this method had a statistical problem that should be avoided, i.e., multicollinearity. The PLS regression model solved the multicollinearity problem by converting the variables into a new set of uncorrelated variables [22]. In addition, modeling with the PLS regression allowed for a machine-learning approach by training the model based on training data; thus, increases in the number of Hanwoo cattle data may enhance the accuracy in the estimated CWT by body measurements. For instance, the data were updated with every slaughter of a Hanwoo cattle in NIAS and elsewhere, and we could use them to train the developed model. However, compared to the multiple regression model, the PLS regression model was relatively complex in terms of model development and interpretation of the results. In other words, the PLS regression model was statistically adequate for the inter-related variables (e.g., body measurements and weight), but it could not improve on model accuracy. This suggested that multicollinearity did not affect the estimation as suggested by previous studies, which used multiple regression analysis without considering collinearity among variables [8–10]. The ANN model was the most modern way to develop a model with advantages in the application of deep learning to improve model prediction. It was important to determine the number of layers and nodes, initial values, and weights through iteration, whereas the statistics-based models required predetermination of significant variables [26]. Like the PLS regression model, it had advantages in that it improved the model by adding newly accumulated data. However, the ANN model was more suitable for a non-linear system than a linear system as shown in the R^2^-value, which was not highly increased compared to statistics-based model (only 1% increase in this study) [29]. Moreover, deep learning by the neural network required a significantly large number of data points; thus, benefits of deep learning by ANN were not been fully exploited in this study. For instance, the ANN would have a great advantage for analyzing images, which provided numerous data points for training and testing of the model [30].

In addition to model structure and accuracy, the number of variables was practically important in this study because variables were hand measured. Hence, an increase in the number of variables could increase the burden of measurement of the body size parameters and increase the possibility of errors. From this point of view, the statistics-based models with only significant variables would be better and the simplest model was more effective than the other types of models. In contrast, when using machine vision, many body measurements could be automatically extracted. Consequently, a large number of variables could be used and the number of data points for training the model could be obtainable. Therefore, a model with high accuracy for estimating CWT by body measurements is possible.

## CONCLUSION

This study estimated CWT as a function of body measurements of Hanwoo cattle by using three different types of modeling approaches. All three modeling approaches showed that body measurements could be applied in the estimation of carcass weight of Hanwoo cattle. In addition, the results suggested that the selected model should be based on numerous variables measured and numerous data points, which are determined by the system (human judgement or machine vision). However, this study was expected to provide a simple algorithm to estimate the carcass weight with reduced labor and time, which was applicable for developing an automated system for Hanwoo cattle measurement. At present, we only used a total of 134 Hanwoo cattle data points, but the model could be updated with the addition of new data. Furthermore, a specific model for estimating carcass weight while considering age and gender and classifying Hanwoo cattle by gender during the application of deep learning are studies to be undertaken in the future [31]. Finally, this study proposes a way to apply recent modeling techniques into animal data analysis, which potentially suggests further applications of them in this field.

## Figures and Tables

**Table 1:** Summary of variable statistics

Value1)	CWT2) (kg)	FT (mm)	EMA (cm^2^)	L (cm)	LF (cm)	LB (cm)	L1 (cm)	L2 (cm)	L3 (cm)	L4 (cm)	L5 (cm)	L6 (cm)	L7 (cm)	L8 (cm)	L9 (cm)	L10 (cm)	L11 (cm)	L12 (cm)	L13 (cm)	L14 (cm)
AVG	429.6	15.2	90.7	257.4	112.7	146.5	44.5	79.1	41.6	34.0	104.2	78.8	78.9	42.9	47.4	173.3	131.1	24.5	21.7	20.0
STD	70.0	7.6	11.2	13.6	6.9	7.2	4.5	4.7	4.1	4.4	10.8	5.4	4.7	3.6	5.7	10.1	9.0	3.1	2.9	3.2
MAX	567.5	40.0	130.0	295.0	133.0	166.0	84.0	97.0	79.0	45.0	130.0	96.0	90.0	53.0	81.0	195.0	182.0	34.0	33.0	38.0
MIN	233.3	1.0	62.0	230.0	97.0	130.0	37.0	65.0	35.0	24.0	27.0	65.0	69.0	32.0	36.0	129.0	110.0	14.0	14.0	13.0
Q1	377.5	10.8	83.0	247.0	108.0	141.0	42.0	76.0	40.0	31.0	102.0	74.0	76.0	41.0	44.0	168.0	126.3	22.0	20.0	18.0
Q3	477.9	19.0	98.3	267.0	117.0	151.0	46.0	82.0	43.0	37.0	109.8	83.0	83.0	45.0	50.0	180.8	135.0	27.0	23.0	21.0

1) AVG, average; STD, standard deviation; MAX, maximum value; MIN, minimum value, Q1: first quartile, and Q3: third quartile.

2) CWT, cold carcass weight; FT, backfat thickness; EMA, eye muscle area; L, side length; LF, forequarter length; LB, hindquarter length; L1, cervical vertebrae length; L2, thoracic vertebrae length; L3, lumbar vertebrae length; L4, sacral vertebrae length; L5, 6th lumbar vertebrae-heel length; L6, 7th cervical vertebrae carcass breadth; L7, 5–6th thoracic vertebrae breadth; L8, 4–5th lumbar vertebrae breadth; L9, 5th sacral vertebrae breadth; L10, 7–8th thoracic vertebrae girth; L11, coxae girth; L12, 4–5th lumbar vertebrae thick; L13, coxae thick; L14, 7–8th thoracic vertebrae thick.

**Table 2:** Results of Pearson correlation coefficients (r) between cold carcass weight and size measurements

Value1)	FT2)	EMA	L	LF	LB	L1	L2	L3	L4	L5	L6	L7	L8	L9	L10	L11	L12	L13	L14
r	0.19	0.69	0.82	0.67	0.73	0.43	0.61	0.30	0.57	0.52	0.83	0.82	0.60	0.55	0.75	0.64	0.46	0.17	0.40
p	0.03	<0.01	<0.01	<0.01	<0.01	<0.01	<0.01	<0.01	<0.01	<0.01	<0.01	<0.01	<0.01	<0.01	<0.01	<0.01	<0.01	<0.01	<0.01
N	132	132	134	134	134	134	134	134	134	134	134	134	134	134	134	134	134	134	134

1) r, p, and N indicates Pearson correlation coefficient, p-value, and number of samples, respectively.

2) FT, backfat thickness; EMA, eye muscle area; L, side length; LF, forequarter length; LB, hindquarter length; L1, cervical vertebrae length; L2, thoracic vertebrae length; L3, lumbar vertebrae length; L4, sacral vertebrae length; L5, 6th lumbar vertebrae-heel length; L6, 7th cervical vertebrae carcass breadth; L7, 5–6th thoracic vertebrae breadth; L8, 4–5th lumbar vertebrae breadth; L9, 5th sacral vertebrae breadth; L10, 7–8th thoracic vertebrae girth; L11, coxae girth; L12, 4–5th lumbar vertebrae thick; L13, coxae thick; L14, 7–8th thoracic vertebrae thick.
